# STAT3 Knockdown Reduces Pancreatic Cancer Cell Invasiveness and Matrix Metalloproteinase-7 Expression in Nude Mice

**DOI:** 10.1371/journal.pone.0025941

**Published:** 2011-10-03

**Authors:** Hai dong Li, Chen Huang, Ke jian Huang, Wei dong Wu, Tao Jiang, Jun Cao, Zhen zhong Feng, Zheng jun Qiu

**Affiliations:** 1 Department of General Surgery, Affiliated First People's Hospital, Shanghai Jiao Tong University, Shanghai, China; 2 Shanghai Key Laboratory of Pancreas Disease, Shanghai, China; 3 Pancreatic Cancer Center of Shanghai Jiao Tong University, Shanghai, China; 4 Department of Pathology, Shanghai Jiao Tong University-Affiliated First People's Hospital, Shanghai, China; University of Illinois at Chicago, United States of America

## Abstract

**Aims:**

Transducer and activator of transcription-3 (STAT3) plays an important role in tumor cell invasion and metastasis. The aim of the present study was to investigate the effects of STAT3 knockdown in nude mouse xenografts of pancreatic cancer cells and underlying gene expression.

**Methods:**

A STAT3 shRNA lentiviral vector was constructed and infected into SW1990 cells. qRT-PCR and western immunoblot were performed to detect gene expression. Nude mouse xenograft assays were used to assess changes in phenotypes of these stable cells in vivo. HE staining was utilized to evaluate tumor cell invasion and immunohistochemistry was performed to analyze gene expression.

**Results:**

STAT3 shRNA successfully silenced expression of STAT3 mRNA and protein in SW1990 cells compared to control cells. Growth rate of the STAT3-silenced tumor cells in nude mice was significantly reduced compared to in the control vector tumors and parental cells-generated tumors. Tumor invasion into the vessel and muscle were also suppressed in the STAT3-silenced tumors compared to controls. Collagen IV expression was complete and continuous surrounding the tumors of STAT3-silenced SW1990 cells, whereas collagen IV expression was incomplete and discontinuous surrounding the control tumors. Moreover, microvessel density was significantly lower in STAT3-silenced tumors than parental or control tumors of SW1990 cells. In addition, MMP-7 expression was reduced in STAT3-silenced tumors compared to parental SW1990 xenografts and controls. In contrast, expression of IL-1β and IgT7α was not altered.

**Conclusion:**

These data clearly demonstrate that STAT3 plays an important role in regulation of tumor growth, invasion, and angiogenesis, which could be act by reducing MMP-7 expression in pancreatic cancer cells.

## Introduction

Pancreatic cancer is a lethal disease and is the fourth most common cause of cancer-related death in Western countries [1]. Most recent data estimated that 43,140 new cases were diagnosed in 2010, with approximately 36,800 associated deaths in the United States [2]. Although surgical resection provides a potential cure, pancreatic cancer is often diagnosed at the advanced stages due to lack of symptoms or nonspecific symptoms, making surgery impossible. Palliative chemotherapy could improve the quality of life and potentially affect survival. These have led to a very dismal prognosis for pancreatic cancer patients, i.e., the median survival is approximately 3 to 6 months and 5-year survival is <5%. Risk factors for pancreatic cancer include smoking, alcohol consumption, diets high in red meat but low in vegetables and fruits, chronic pancreatitis, and family history. However, the molecular mechanisms responsible for pancreatic cancer development have not been elucidated and there are no established guidelines for pancreatic cancer prevention. Therefore, novel approaches to diagnose pancreatic cancer early to ensure effective therapy are drastically needed.

The signal transducer and activator of transcription (STAT3) gene is a member of the STAT protein family of transcription factors. In response to cytokines and growth factors, STAT family members are phosphorylated by receptor-associated kinases, forming homo- or heterodimers and translocating in the nucleus of cells to bind to DNA and regulate expression of the target genes. STAT3 is activated via phosphorylation of tyrosine 705 and serine 727 in response to various cytokines and growth factors (such as interferon, EGF, and interleukins). STAT3 plays an important role in mediating various cellular processes (e.g., cell growth, apoptosis, and differentiation) [3]. Previous studies have shown that the oncogenic STAT-3 protein was constitutively activated in many human cancers [4]–[6]. In contrast, inhibition of the STAT-3 signaling pathway suppressed cancer cell invasion in various cancers [7]–[9]. In pancreatic cancer, activation of STAT-3 promoted tumor cell growth, invasion, and metastasis, leading to poor patient survival. Molecularly, STAT3 can regulate expression of VEGF and MMP-2 genes [10]–[11]. These two genes, together with MMP-7, MMP-9, bFGF, IL-1β and IgT7αα are closely related to tumor progression (angiogenesis, invasion and metastasis) [12]–[14]. In the present study, we utilized the STAT3 shRNA lentivirus to silence STAT3 expression. Previous data from our group suggests that knockdown of STAT3 inhibited invasion of pancreatic cancer SW1990 cells *in vitro* and markedly decreased VEGF and MMP-2 expression [7]. In the present study, we investigated whether silencing of STAT3 in pancreatic cancer cells modulates tumor cell growth and invasiveness in nude mouse xenografts and determined the underlying signaling mechanisms involved using a cDNA microarray.

## Materials and Methods

### Cell culture and lentivirus infection

The human pancreatic cancer cell line SW1990 was obtained from American Type Culture Collection (Manassas, VA, USA) and cultured with Dulbecco's modified Eagle's medium (DMEM) supplemented with 10% fetal bovine serum (FBS) and penicillin/streptomycin at 37°C in a 5% CO_2_ and 95% air incubator. To silence STAT3 expression, we constructed a lentiviral vector carrying STAT3 shRNA as described previously [7]. SW1990 cells (1×10^5^) were seeded into each well and grown in 6-well plates and then infected with a negative control lentiviral vector (Genechem, Shanghai, China) or the lentiviral vector carrying STAT3 shRNA at a multiplicity of infection (MOI) of 40. After three days, the cells were subjected to *vivo* invasion assay.

### Quantitative real-time reverse transcription-polymerase chain reaction (qRT-PCR)

Total RNA was isolated from parental SW1990 and negative control vector or STAT3 vector-infected SW1990 cells using Trizol reagent from Invitrogen (Carlsbad, CA, USA). The RNA was then purified using an RNeasy mini kit according to the manufacturer's instructions (Qiagen, Valencia, CA, USA). Following quantification, 1 µg of total RNA from each sample was subjected to first-strand cDNA synthesis using a PrimeScript RT Reagent kit (TaKaRa Bio Inc, Shiga, Japan) at 37°C for 15 min and 85°C for 5 s.

qPCR was then performed to detect gene expressions with these newly synthesized cDNA. The specific primers for the PCR reaction were as follows: MMP-9, 5′-CGGAGTGAGTTGAACCAG-3′ (forward) and 5′-GTCCCAGTGGGGATTTAC-3′ (reverse) with a product size of 118 bp; MMP-7, 5′-GAGTGCCAGATGTTGCAGAA-3′ (forward) and 5′-AAATGCAGGGGGATCTCTTT-3′ (reverse) with a product size of 169 bp; IL1-β, 5′-CTGAGCTCGCCAGTGAA-3′ (forward) and 5′-GGTCTGTGGGCAGGGAA-3′ (reverse) with a product size of 239 bp; bFGF, 5′-AGAGCGACCCTCACATCAAG-3′ (forward) and 5′-TCGTTTCAGTGCCACATACC-3′ (reverse) with a product size of 224 bp; IgTα7, 5′-GCGGCCACTCGGTCTGTGTGGAC-3′ (forward) and 5′-GGAGACTGTAGGACAAGGTCAC-3′ (reverse) with a product size of 303 bp; β-actin, 5′-TCACCCACACTGTGCCCATCTACGA-3′ (forward) and 5′-CAGCGGAACCGCTCATTGCCAATGG-3′ (reverse) with a product size of 294 bp. qPCR amplifications were performed using the DNA Engine Opticon System (Bio-rad, Foster city, CA, USA) with SYBR Premix Ex Taq (TaKaRa). Specifically, 1 µl of reverse transcription reaction mixture was utilized for a quantitative PCR reaction in a total volume of 20 µl. PCR cycles were 95°C for 30 s followed by 40 cycles of 95°C for 5 s and 60°C for 30 s. These data were then analyzed according to the comparative Ct method and normalized to β-actin expression levels within each sample. Relative expression levels of target genes, following normalization to an endogenous sequence, were given by 2^−ΔΔ*Ct*^.

We also performed PCR array experiments in an RT^2^ Profiler Human Tumor Metastasis PCR Array (PAHS-028A) from SuperArray Bioscience (Frederick, MD, USA). This array contains 89 genes and five “housekeeping genes” in a 96-well plate. We utilized this array to detect knockdown of STAT3-mediated gene expression according to the manufacturer's protocol. Each reaction included 40 ng of total RNA and the proper negative controls (no reverse transcription and no template). Samples were analyzed in triplicate, and the data were normalized to GAPDH levels using the ΔΔCt method.

### Protein extraction and Western immunoblot

Cells were lysed in radioimmunoprecipitation assay buffer (Beyotime, Haimen, Jiangsu, China) containing 1 mmol/L phenylmethanesulfonyl fluoride on ice for 15 min. Protein concentration was determined using a BCA protein assay kit (Beyotime). Lysates were then mixed with SDS-PAGE sample loading buffer and boiled for 5 min. 30 µg of total cellular protein was then resolved on 8% or 10% SDS-polyacrylamide gels and transferred onto nitrocellulose membranes. The membranes were then stained with 0.5% Ponceau S containing 1% acetic acid in order to assess for equal loading and transfer efficiency. The membranes were then blocked in 5% bovine skim milk overnight and with the primary antibody for 4 h at room temperature. Following washing in phosphate buffered saline (PBS), the membranes were further incubated with a peroxidase-conjugated secondary antibody for 1 h at room temperature. To detect positive protein bands, the enhanced chemiluminescence reagent from Millipore (Billerica, MA, USA) was used. The primary antibodies were as follows: MMP-7 from R&D Systems (Minneapolis, MN, USA) at a concentration of 2 µg/ml, MMP-9 from Epitomics (Burlingame, CA, USA) at a dilution of 1∶2000, bFGF, IL1-β and IgTα7 from Santa Cruz Biotechnology (Santa Cruz, CA, USA) at a dilution of 1∶200, STAT-3 from Cell Signaling Technology (Danvers, MA, USA) at a dilution of 1∶1000; and β-actin from Biomart (Shanghai, China) at a dilution of 1∶1000. Secondary antibodies included peroxidase-conjugated affinipure goat anti-mouse or anti-rabbit IgG from Jackson ImmunoResearch (West Baltimore Pike West Grove, PA, USA).

### Nude mouse experiments

Six week old SPF grade male BALB/c nude mice were purchased from the Institute of Zoology, Chinese Academy of Sciences (Shanghai, China). These mice were fed a standard rodent diet and water *ad libitum* in an aseptic laminar flow room with 60%-70% humidity at 25°C. Two weeks following arrival, 50 µl cell solutions (containing 2×10^6^ logarithmic growth phase tumor cells) were injected into the nail pad of the mice subcutaneously. The mice were then observed daily for diet consumption, stools and mental state, and the tumor size and body weight were measured every five days. The length and width of the tumor were measured with Vernier calipers and calculated using a formula of tumor volume  =  length×width^2^×0.5. The use of animals in this study compiles with the Guide for the Care and Use of laboratory Animals. This study was approved by Science and Technology Commission of Shanghai Municipality (License #SYXK2009-0086).

### Immunocytochemistry and immunohistochemistry

Tumor cells grown on glass coverslips were washed with PBS in triplicate and fixed with 4% formaldehyde. Endogenous peroxidase activity from the cells was blocked with 3% hydrogen peroxide. The coverslips were then incubated with 1% bovine serum albumin in PBS for 1 h at room temperature and with primary antibody overnight at 4°C, followed by incubation with a secondary antibody for 30 min. The coverslips were then counterstained with Haematoxylin solution.

For immunohistochemical staining, 4 µm sections were cut from formalin-fixed paraffin-embedded tissue blocks and then deparaffinized in xylene and rehydrated in successive washes of ethanol. The sections were then heated in a microwave oven at medium power for 8 min in citrate buffer, pH 6.0 for heat-induced epitope retrieval. The sections were subjected to blockade of endogenous peroxidase activity and non-specific binding of the primary antibody and then target protein localization with the first antibody and visualization with the secondary antibody and color reaction as described above.

The primary antibodies included: MMP-7 from R&D Systems at a concentration of 10 µg/ml; collagen IV or PECAM-1 from Bioworld Technology (Louis Park, MN, USA) at a dilution of 1∶100. The secondary antibodies were goat anti-mouse or anti-rabbit IgG conjugated with peroxidase from DAKO EnVision (DAKO Corp., Carpinteria, CA).

Stained tumor cells and paraffin sections were reviewed and scored using a light microscope by a pathologist blinded to the treatment group. Positivity of the stained tumor cells on coverslips and paraffin sections were defined by staining intensity and % of tumor cells: the staining intensity of MMP-7 expression was classified semiquantitatively into negative and weak, moderate, and strongly positive (0, +, ++, and +++, respectively). In our study, gene expression was considered positive if the staining intensity was moderate or strong and the percentage of positive stained cells were >20%. However, the density of microvessels staining positive for PECAM-1 was defined as positive at 400 power of a microscope field as described previously [15].

### Statistical Analyses

Statistical analyses were performed using SPSS 12.0 software (SPSS, Chicago, IL, USA). The data were summarized as mean ± SD when possible and analyzed using a Student-Newman-Keuls test to determine statistical significance. P<0.05 was considered statistically significant.

## Results

### Knockdown of STAT3 expression using STAT3 shRNA

**Figure 1:**
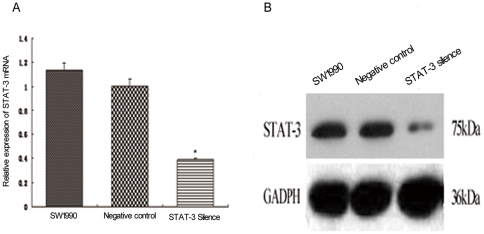
Suppression of STAT3 mRNA and protein expression using STAT3 shRNA in pancreatic cancer SW1990 cells. A, qRT-PCR. Stable STAT3 shRNA and control vector-transfected SW1990 cells and parental SW1990 cells were grown and RNA was isolated for qRT-PCR analysis of STAT3 mRNA expression *P<0.01 compared to SW1990 or control vector-transfected cells. B, Western immunoblot. These cells were grown for total cellular protein extraction and western immunoblot analyses of STAT3 protein expression.

To demonstrate the role of STAT3 in pancreatic cancer, we performed gene knockdown experiments using STAT3 shRNA in SW1990 cells. We determined that STAT3 shRNA successfully knocked down STAT3 expression in SW1990 cells, i.e., qRT-PCR data showed that STAT3 shRNA vector inhibited STAT3 mRNA expression compared to control vectors (*P* = 0.004), while western immunoblotting data showed the STAT3 protein was markedly inhibited in STAT3 shRNA-transfected SW1990 cells (*P* = 0.003) (Figure 1).

### Suppression of growth and invasion of STAT3 shRNA-transfected SW1990 cells in nude mice

**Figure 2:**
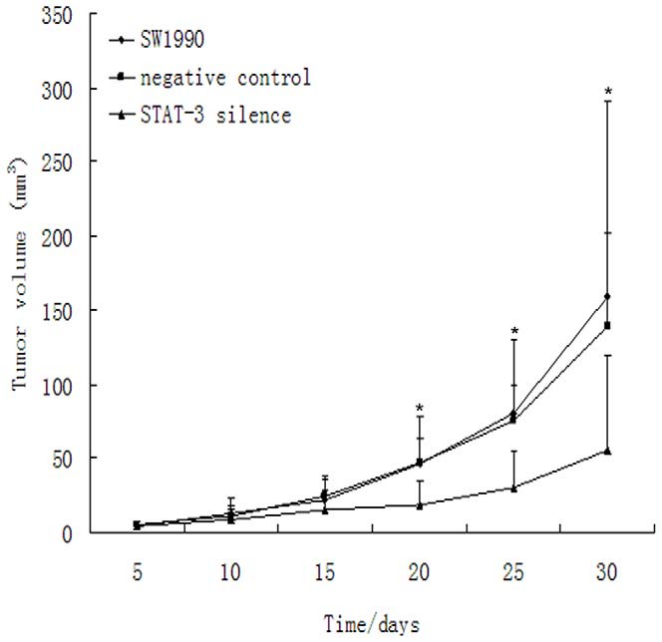
Suppression of nude mouse xenograft growth by STAT3 knockdown. Parental or control vector and STAT3 shRNA-transfected SW1990 cells were grown in cell culture and injected into nude mouse nail pads. Tumor volume was measured every five days up to 30 days following tumor cell inoculation.. *P<0.05 compared to the control vector or parental tumors.

**Figure 3:**
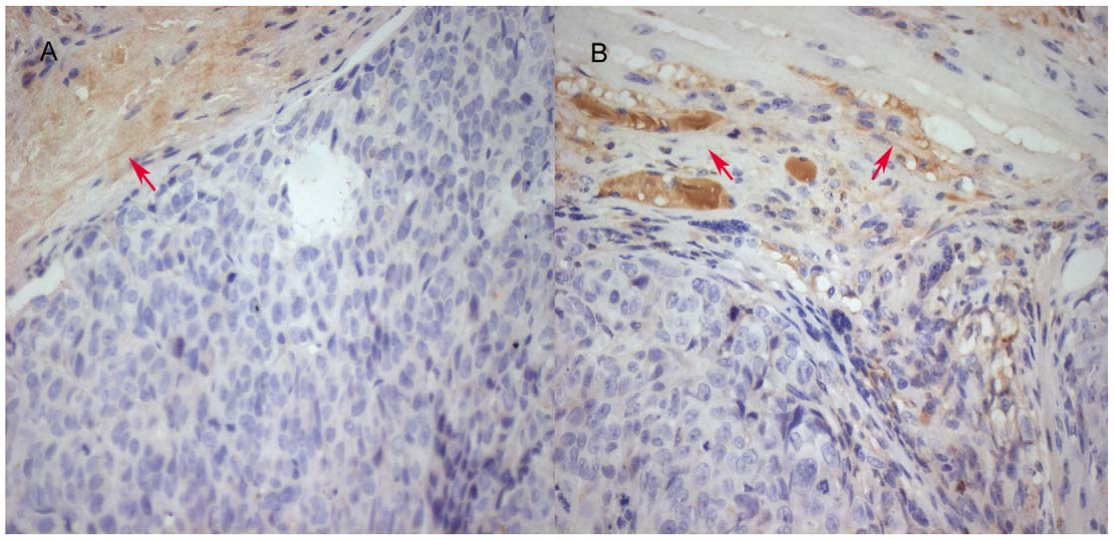
Expression of collagen IV protein surrounding the tumor cells using immunohistochemistry. The tumor xenografts from control vector and STAT3 shRNA-transfected cells were subjected to immunohistochemical staining of collagen IV protein. These data suggest that collagen IV expression (red arrow) is complete and continuous in STAT-3-silenced tumor (A), however the integrity of collagen IV was destroyed evidenced by incomplete and discontinuous (red arrow) staining (B).×400 magnification.

**Figure 4:**
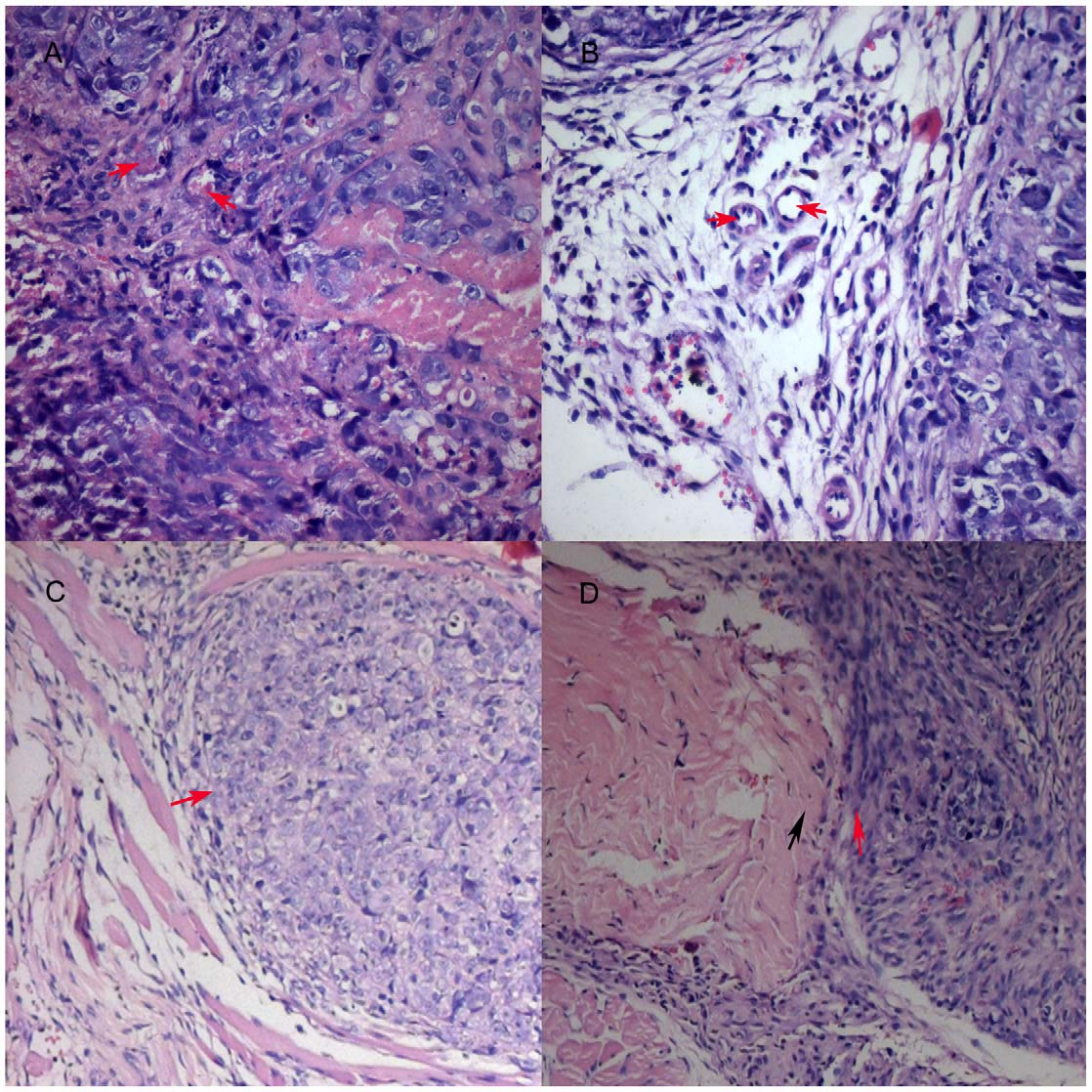
Suppression of tumor invasion in nude mouse xenografts by STAT3 shRNA. These nude mouse xenografts were taken from tissues processed for HE staining. A and B, x 400 magnification, C and D, x 100 magnification. Tumor cell invasion into the microvessel is frequent in control vector tumors (marked in red arrow in A) but not obvious in STAT3-silenced tumors (B). The muscle was invaded and destroyed (black arrow) by the tumor cells (red arrow) of SW1990 cells in C, but the boundary is clear between muscle (black arrow) and tumor (red arrow) in D.

**Figure 5:**
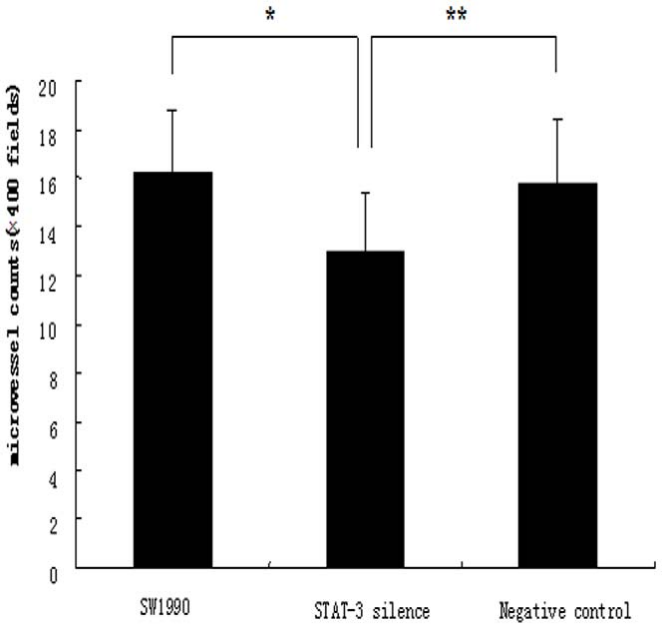
Suppression of microvessel density by STAT3 shRNA in nude mouse xenografts. Tissue sections of nude mouse xenografts were stained for immunohistochemistry with the anti-PECAM-1 antibody. PECAM-1 positive microvessels in tumors were counted under a microscope and summarized. The number of microvessel was 13.00±2.36 per high power field (N = 9) in STAT3-silenced tumors compared to 16.30±2.45 (N = 10) in the parental SW1990 tumors and 15.80±2.57 (N = 10) in vector control tumors. These differences were statistically significant. *P<0.01 vs. parental SW1990 tumors, **P<0.05 vs. the control vector tumors.

We injected stable STAT3 knockdown or control cells into the nail pad of nude mice. 10 days following tumor inoculation, we found that growth rate of the STAT3-silenced tumor cells was significantly decreased (Figure 2). We also found that expression of collagen IV protein surrounding the tumor cells in control tumors was incomplete and discontinuous, whereas it was complete and continuous in tumors of STAT3-silenced SW1990 cells following immunohistochemical staining (Figure 3A & B). Tumor cell invasion ability was detected on HE-stained tissue xenografts by light microscope. Furthermore, tumor invasion into the vessel and muscle was more frequent in the control tumors than STAT3-silenced tumors (Figure 4). The percentage of vessel and muscle invasion by STAT3-silenced tumors was 22.22% (2/9) and 33.33% (3/9) compared to 60% (6/10), 80% (7/10) and 70% (7/10), 80% (8/10) in SW1990 tumor and negative control tumor, respectively. Microvessel density was significantly lower in STAT3-silenced tumors than parental or control tumors of SW1990 cells (*P* = 0.007 and *P* = 0.021, respectively; Figure 5). These data indicate that STAT3 plays a crucial role in the regulation of tumor growth, invasion, and angiogenesis.

### Expression of tumor invasion and metastasis genes following STAT3 knockdown

We determined gene expression in these STAT3-knocking down tumors using a human tumor metastasis PCR array (#PAHS-028A) from SuperArray Bioscience. PCR array data suggest that three genes, i.e., MMP-7, IL-1β and IgT7α, were downregulated (reductions of 2.54-fold, 23.90-fold, and 5.39-fold, respectively) in STAT3-silenced tumors compared to the control and parental SW1990 tumors. However, eight genes were upregulated, : SYK (3.38 folds), MYCL1 (4.85 folds), MMP-11 (4.59 folds), MMP-3 (10.88 folds), MMP-10 (19.93 folds), CST-7 (3.34 folds), CDH11 (2.01 folds), and IL-8Rβ (7.06 folds).

### Reduced MMP7 mRNA and protein expression in STAT3-silenced tumors

**Figure 6:**
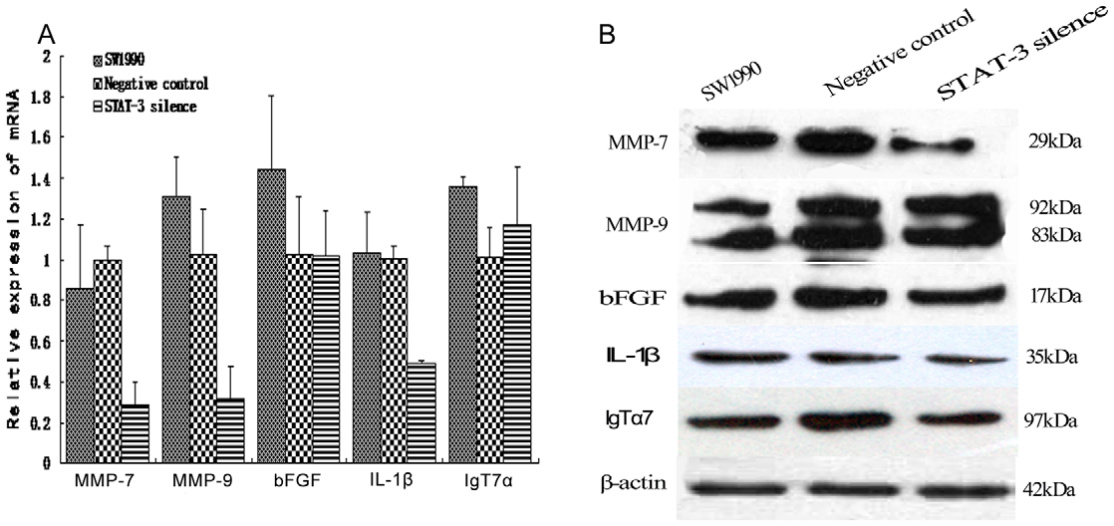
Expression of MMP-7, MMP-9, bFGF, IL-1b, and IgT7α in nude mouse xenografts. A, qRT-PCR. RNA was isolated from the tumor xenografts and subjected to qRT-PCR analyses. B, Western blot. Total cellular protein was extracted from the nude mouse xenografts and subjected to western blot analyses. **P<0.01; *P<0.05 compared to the control vector or parental tumors, respectively.

We validated data from the array experiments and performed additional analyses using qRT-PCR (Fig. 6A & S1) and western immunoblotting (Figure 6B) and found that MMP-7, MMP-9 mRNA expression was significantly decreased in STAT3-silenced tumors compared to parental and control tumors (*P* = 0.033 and *P* = 0.002 *vs.* SW1990, respectively or *P* = 0.0001 and *P* = 0.011 *vs.* control tumors, respectively). IL-1β mRNA expression was also significantly reduced in STAT3-silenced tumors compared to parental and control tumors (*P* = 0.01 and P = 0.0001 *vs.* SW1990 and control tumors, respectively). However, there was no significant difference in bFGF or IgT7α mRNA expression between the STAT3-silenced tumors or controls (Fig. 6A). MMP-7 expression at the protein level was markedly suppressed in STAT3-silenced tumors, however MMP-9 was not significantly affected (Figure 6B).

### Reduced MMP-7 expression in STAT3-silenced tumor cells and xenografts

**Figure 7:**
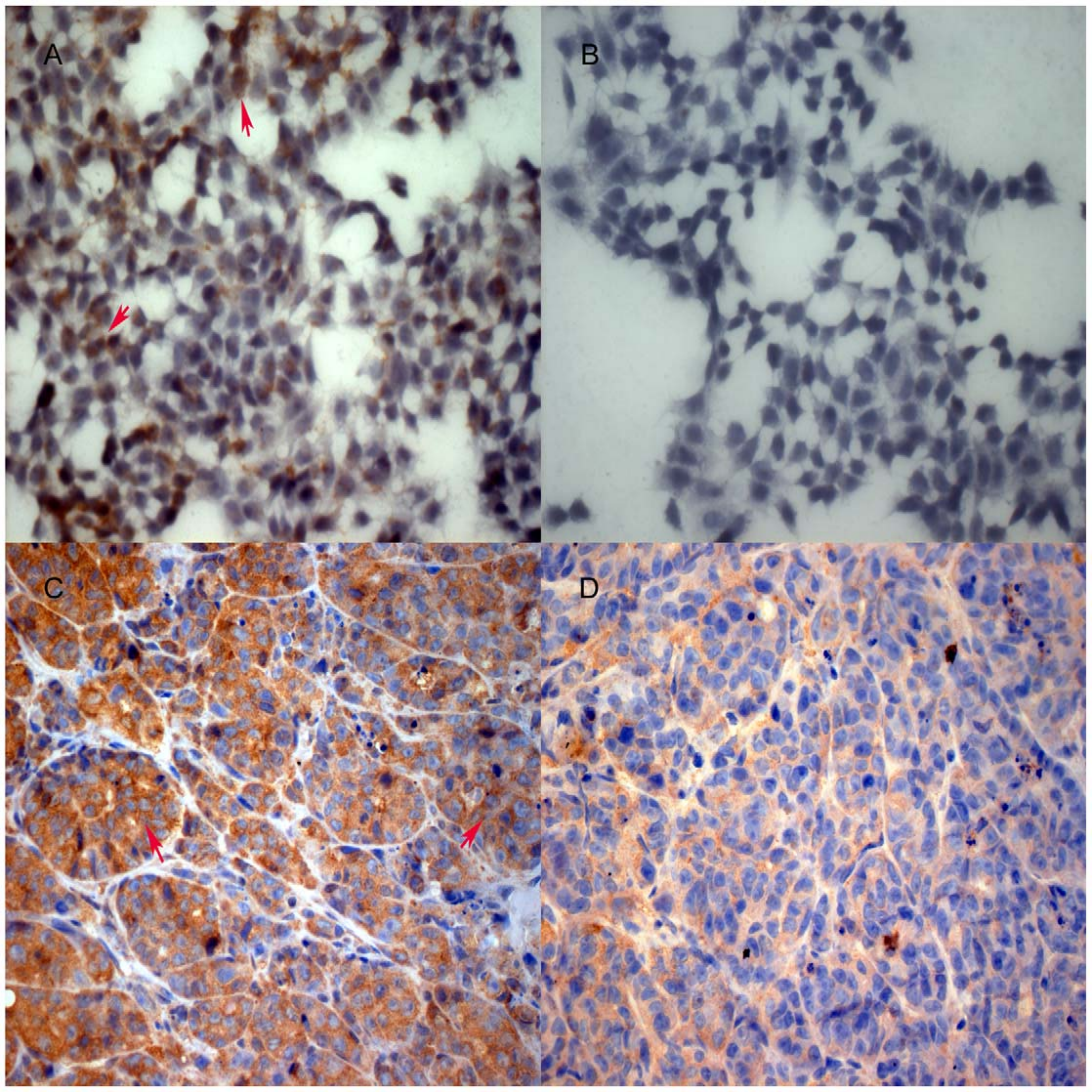
MMP-7 protein expression in pancreatic cancer cells in monolayer and in nude mouse xenografts. Tumor cells from parental, vector control and STAT3-silenced SW1990 cells were grown in a monolayer or in nude mice. The monolayer cells and tumor xenografts were then subjected to immunocytochemistry and immunohistochemistry staining of MMP-7 protein. A, parental cells; B, STAT3-silenced cells; C, parental tumors; D, STAT3-silecnced tumors. X 400 magnification. Red arrow, tumor cells.

To confirm the above data, we performed immunocytochemistry on the STAT3 knockdown cells and nude mouse xenografts. Our data suggest that STAT3-silenced SW1990 cells or xenografts expressed lower levels of MMP-7 protein in the cytoplasm compared to the parental and control cells and xenografts (Figure 7), which is consistent with the invasion restraint in nude mouse xenografts. These data indicate that STAT3 regulation of invasion capacity in pancreatic cancer cells is associated with downregulation of MMP-7 expression.

## Discussion

STAT3 shRNA successfully knocked down expression of STAT3 mRNA and protein in SW1990 cells compared to control vector-transfected SW1990 cells. We then subcutaneously injected the control vector and STAT3 shRNA-transfected SW1990 cells into nude mice. Our data suggest that growth rate of the STAT3-silenced tumor cells in nude mice was significantly reduced compared to control vector cells. Tumor invasion into the vessel and muscle was more infrequent in the STAT3-silenced tumors than in control tumors. Expression of collagen IV protein surrounding the tumor cells was complete and continuous in tumors of STAT3-silenced SW1990 cells, whereas collagen IV expression was incomplete and discontinuous within control tumors. Furthermore, microvessel density was significantly lower in STAT3-silenced tumors than parental and control tumors of SW1990 cells. At the molecular level, MMP-7 expression was reduced in STAT3-silenced tumors compared to the control and parental SW1990 xenografts. Data from the current study clearly indicate that STAT3 does play a crucial role in the regulation of tumor growth, invasion, and angiogenesis.

However, we did not find significant changes in MMP-9 protein expression, although expression of MMP9 mRNA was reduced. The reason for this discrepancy may be due to tissue inhibitor of metalloproteinases 1 (TIMP-1) decreased when STAT3 silencing led to activated MMP-9 increases, which decreased expression of MMP9 mRNA through negative feedback [16], [17]. Moreover, our current data also suggests some discrepancies between PCR array and qRT-PCR data. Indeed, PCR array is a novel technique to investigate differential expression of target genes at the mRNA level with high throughput, sensitivity and specificity. However, these data always need to be confirmed using qRT-PCR, some of which may be verified (confirmed) using qPCR. This reason may be due to design of the primer and difficulty in controlling the annealing temperature leading to non-specific amplifications in PCR array. Thus, qRT-PCR data could be more specific and reliable. Although we have shown some genes that were upregulated after silencing of STAT3 expression, we would like to emphasize the genes that were downregulated following STAT3 silence in the current study.

STAT3 is a key transcription factor that is oncogenic in human cells [18], [19]. The Janus kinase (JAK)/STAT3 signaling plays an important role in the regulation of cell growth and differentiation and tumor invasion and metastasis in diverse human cancers [20]–[22]. In pancreatic cancer, recent studies have demonstrated inappropriate and constitutive activation of STAT3 [23], [24]. In a previous study by our group, we reported down-regulation of STAT3 expression suppressed invasion capacity of pancreatic cancer cells *in vitro* [7]. In this study, we found that silencing of STAT3 expression suppressed tumor growth and invasion and the microvessel density in nude mice. These data are consistent with a previous *ex vivo* study [25] where the authors demonstrated that expression of phosphorylated STAT3 in human gastric carcinoma significantly correlated with tumor invasion and prognosis *ex vivo*.

Previous studies by others and us have shown that STAT3-increased microvessel density may be due to STAT3 induction of VEGF expression [7], [26]. Another study also provided evidence that suppression of invasion and metastasis by STAT3 knockdown depended on VEGF down-regulation of colon cancer cells [27]. Furthermore, decreased expression of MMP-2 is a key reason for suppression of tumor cell invasion and metastasis following silencing of STAT-3 expression in human melanoma [5]. MMPs play important roles in tumor cell invasion and metastasis by the degradation of components of basement membranes and extracellular matrix [28]–[30]. An increasing number of studies indicate that certain MMP proteins are targeted by STAT3 [31], [32]. Xie et al determined that STAT3 activation promoted invasion of melanoma cells through the regulation of MMP-2 gene transcription [33]. In addition, MMP-1 and MMP-9 were found to be regulated by STAT3, which plays a crucial role in tumor invasion and metastasis [34], [35]. In the current study, we showed that knockdown of STAT3 expression decreased MMP-7 expression in pancreatic cancer cells and nude mouse xenografts. MMP-7 is the smallest protein in the MMP family but possesses the highest extracellular matrix (ECM)-degradative activity against a variety of ECM components; thus, it is capable of triggering the activation of an MMP cascade and is related closely to tumor invasion and metastasis [36]–[39]. In pancreatic cancer, MMP-7 has been shown to be involved in tumor cell dissociation from the original site and subsequently gain invasion capacity [40], [41]. Other studies have suggested that MMP-7 could be a direct target for STAT3 in gastric and breast cancer cells [42], [43], which is consistent with our conclusion in pancreatic cancer cells.

In the current study, we utilized the nude mouse nail pad model instead of the routine nude mouse flank model of tumor cells. The advantage of the nail pad model is that this model tests not only the invasion ability of tumor cells into the vessel and muscle, but also a certain method of inguinal lymph nodes metastases of tumor cells. Nevertheless, the orthotopic tumor model is the best for detecting invasion and metastasis ability of tumor cells. In pancreatic cancer, we faced several difficulties, including the technique to inject tumor cells into the pancreas, surgery inducing high mortality, and the uncertainty of tumor invasion and metastases (different from the primary tumors in the pancreas). In this context, the nude mouse nail pad model is easy and reliable. These current data suggest that STAT3 knockdown significantly altered invasion ability of the pancreatic cancer cells, without any metastatic tumors in the lymph nodes in both experimental and control groups. This may be due to the time period of the experiments (30 days). This study also demonstrated the effects of STAT3 knockdown on pancreatic cancer cells *in vivo*, which confirmed our previous *in vitro* data that STAT3 plays an important role in promoting tumor growth, invasion, and angiogenesis, whereas suppression of STAT3 expression did inhibit pancreatic cancer cell growth, angiogenesis, and invasion. These functions of STAT3 may act through regulation of its downstream genes, including cyclinD1, Bcl-2, VEGF, and MMP-2, all of which were mentioned in previous studies [26], [44], [45]. In ours study, we found that STAT3 inhibition can reduce MMP-7 expression in pancreatic cancer cells. Future studies should focus on whether silencing of STAT3 expression using STAT3 shRNA or its inhibitor such as non-receptor tyrosine kinase is useful as a novel adjuvant therapy to chemotherapy for pancreatic cancer patients.

## Supporting Information

Figure S1 **qRT-PCR analysis of SYK, MYCL1, MMP-11, MMP-3, MMP-10, CST-7, CDH11, and IL-8Rβ expression in nude mouse xenografts.** RNA was isolated from the mouse tumor xenografts and subjected to qRT-PCR analysis. The data showed that expression of MYCL1, MMP-3, MMP-10 and IL-8Rβ was up regulated, but SYK was down-regulated in STAT3 silence tumor compared to the control vector or parental tumors. In contrast, expression of CST-7, CDH11, MMP-11 mRNA was no difference in each tumor sample.
(TIF)
