# Supplementary figures and images for: STAT3 Knockdown Reduces Pancreatic Cancer Cell Invasiveness and Matrix Metalloproteinase-7 Expression in Nude Mice
Source: PLoS One. 2011 Oct 3;6(10):e25941. doi: 10.1371/journal.pone.0025941 (PMC3185063; doi:10.1371/journal.pone.0025941)

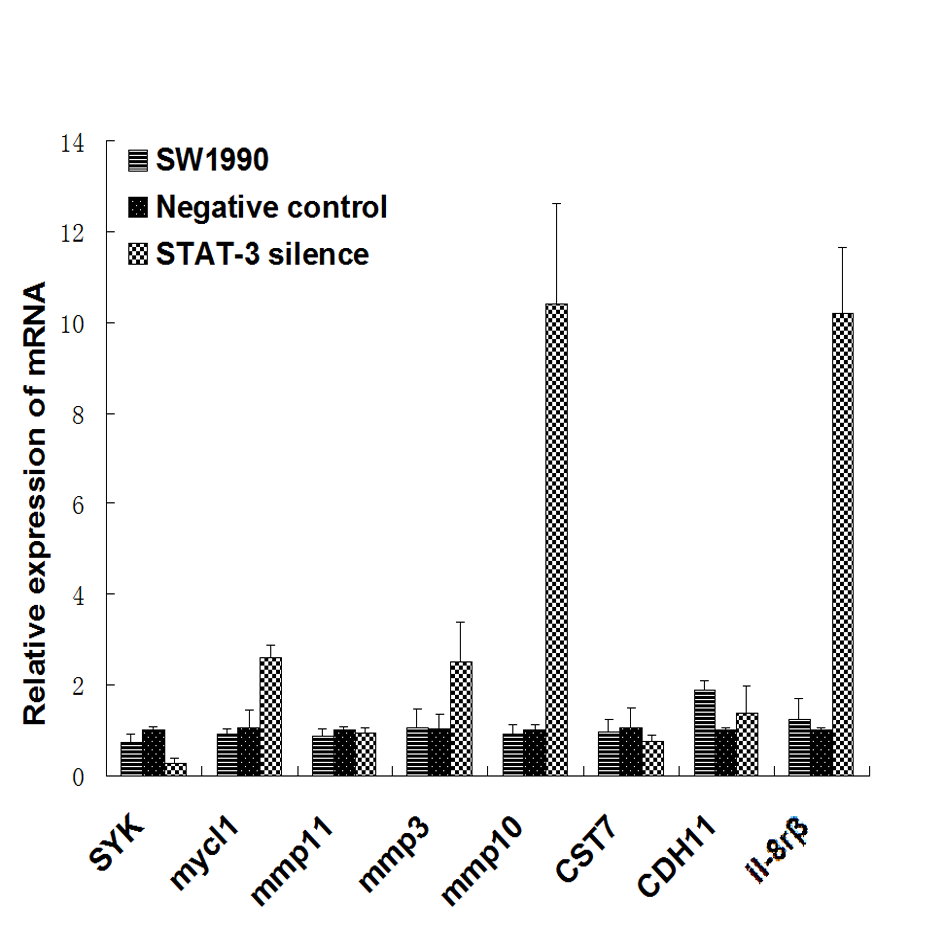

Supplement: Figure S1 — qRT-PCR analysis of SYK, MYCL1, MMP-11, MMP-3, MMP-10, CST-7, CDH11, and IL-8Rβ expression in nude mouse xenografts. RNA was isolated from the mouse tumor xenografts and subjected to qRT-PCR analysis. The data showed that expression of MYCL1, MMP-3, MMP-10 and IL-8Rβ was up regulated, but SYK was down-regulated in STAT3 silence tumor compared to the control vector or parental tumors. In contrast, expression of CST-7, CDH11, MMP-11 mRNA was no difference in each tumor sample. (TIF) [file pone.0025941.s001.tif]
